# Navigating family life in the face of parental cancer: a qualitative study

**DOI:** 10.1007/s00520-026-10670-6

**Published:** 2026-04-22

**Authors:** Gerdien van der Zwaag, Henriëtta J. Bragt-de Jong, Melanie P. J. Schellekens, Marije L. van der Lee

**Affiliations:** 1 Scientific Research Department, Centre for Psycho-Oncology, Helen Dowling Institute, Professor Bronkhorstlaan 20, Bilthoven, 3723MB The Netherlands; 2 Department of Medical and Clinical Psychology, School of Social and Behavioral Sciences, Tilburg University, Tilburg, The Netherlands

**Keywords:** Family functioning, Parental cancer, Autonomy, Connectedness, Communication

## Abstract

**Purpose:**

Parental cancer can significantly impact family dynamics, particularly for adolescents and young adults (AYAs), who face the developmental task of balancing autonomy and connectedness. As cancer incidence rises globally, more AYAs are confronted with the challenges of parental illness. While most research focuses on individual psychological outcomes, less is known about how families function as a unit under such stress. This study aimed to gain deeper insight into family functioning, with a specific focus on autonomy, connectedness, and communication, in the face of parental cancer.

**Methods:**

We conducted an exploratory qualitative study using thematic analysis. Semi-structured in-depth interviews were conducted with eight families (13 parents, 14 AYAs) in which one parent had been diagnosed with cancer. The interview design included separate interviews with parent(s), child(ren), and a joint family interview.

**Results:**

Thematic analysis yielded three main themes: *children’s struggle between personal space and family life*, *barriers and opportunities in family communication*, and *redefining connectedness*. These themes illustrate how families attempt to balance autonomy and connectedness and the role of communication in this.

**Conclusion:**

Families facing parental cancer develop different ways to balance autonomy and connectedness. In some families, strong emotional attunement naturally supported this balance, even in the absence of open communication. Others struggled more with these dynamics. Practical strategies such as maintaining meaningful activities, doing activities that foster open communication, humor, and involving children in the medical process can support family resilience. When emotional attunement does not arise naturally, family-based interventions may help strengthen these processes.

**Supplementary Information:**

The online version contains supplementary material available at 10.1007/s00520-026-10670-6.

## Introduction

Family dynamics change when a parent is diagnosed with cancer, affecting not only the patient, but also their partner and children. Vissers et al. [1] report in their literature review that children whose parent is diagnosed with cancer often exhibit behavioral problems, internalizing problems, and disturbed physical and cognitive functioning. Much of this research has focused on younger children, while far less is known about adolescents and young adults (AYAs, defined as individuals aged 15–29 years). These psychosocial outcomes are influenced by family functioning (i.e., the quality of communication, cohesion, and conflict within the family), as worse family functioning is associated with more psychosocial problems for AYAs [2, 3]. As global cancer incidence rises due to aging and population growth, more AYAs face parental cancer [4]. Lifetime prevalence estimates indicate that between 1.6% and 8.4% of AYAs have a parent with a history of cancer, underscoring the increasing relevance of this issue [5]. In addition, population-based data from Western Australia indicate that 0.47% of AYAs experienced a parent’s cancer diagnosis over a 33-year period [6].

Parental cancer during the AYA years can be particularly challenging due to the dynamic nature of this life stage for children, characterized by significant cognitive, social, and emotional development resulting in a growing autonomy over one’s own life [2]. Specifically, the AYA tries to establish an individual identity, form intimate relationships outside one’s family, pursue a career, and gain financial independence [7]. In this phase, balancing growing autonomy and family connectedness is essential for a healthy identity formation [8]. However, a parental cancer diagnosis could impede this process of balancing autonomy and connectedness, as role shifts, caregiving responsibilities, and emotional strains may undermine autonomy and connectedness [9, 10].

Family resilience plays a crucial role in navigating the challenges that come with a cancer diagnosis and is defined as ‘the capacity of the family system to withstand and rebound from adversity, strengthened and more resourceful’ [11]. The family resilience framework highlights strengths under stress, in response to crisis, or with prolonged adversity. It includes three key processes for family resilience: (1) family belief systems, including making meaning of adversity; (2) organizational processes, including connectedness and autonomy; (3) communication, including open emotional expression. This study focuses on connectedness, communication, and autonomy. Connectedness involves mutual support, collaboration, and commitment among family members, while also respecting AYAs’ autonomy, including their individual needs, differences, and boundaries. Communication refers to family members sharing their emotions, making sense of painful experiences, and preventing isolation. Communication is also a key factor of family connectedness, with lower levels of adolescent-parent communication being linked to reduced family cohesion in the context of adolescents with cancer [12].

While most of the research has focused on the individual impact of cancer on the patient, spouse, and child, one area that has received little attention is the impact of parental cancer on the family dynamics. In-depth insight into a family’s strengths and abilities is essential for preventing mental health problems and supporting families dealing with cancer. The main aim of the present study was to gain a deeper understanding of family functioning, and in particular autonomy, connectedness, and the role of communication, in families with AYAs aged 15–29 facing parental cancer. Qualitative research can help identify the adaptive strategies of families with parental cancer.

## Method

### Study population

Families were eligible if at least one parent had a cancer diagnosis and had at least one child aged 15–29 at the time of participation. Participation required both the ill parent (current or past diagnosis) and at least one child from the same family. Participants also needed to be able to speak and read Dutch.

### Procedures

Recruitment took place from December 2023 to November 2024 via social media and healthcare professionals at the Helen Dowling Institute (HDI), a mental healthcare center for psycho-oncology. Interested families received an information letter and informed consent by mail. After signed consent, participants completed a short socio-demographic and clinical questionnaire, after which interviews were scheduled, consisting of separate parent(s)-only, child(ren)-only, and joint family interviews. These semi-structured interviews were conducted by the first author (GZ; female researcher with clinical experience), two HDI therapists (female and male), or two female research assistants (one MSc graduate, one MSc student in Medical Psychology). Where possible, interviews with parent(s) and child(ren) were held before the family interview. Interviews took place at participants’ homes (*n* = 2) or online (*n* = 19), lasted 33–99 min, and were audio-recorded. To ensure anonymity, families were labeled using letters A to H, assigned sequentially in order of interview.

The interview schedule was based on Walsh’s family resilience framework [11] and developed with input from family therapists from HDI (see Table 1 for the interview guide). Following a brief introduction to the interview, participants were asked questions about autonomy, connectedness, and communication. Each interview concluded with a question to share advice for other families. Joint family interviews built on themes from individual sessions, capturing real-time family dynamics.

This study was approved by Tilburg University’s ethics review board under reference number RP1049.

**Table 1:** Guide for semi-structured interviews

Topic	Parent interview	AYA interview	Family interview
Opening	How did you feel when you heard the diagnosis? How did you discuss this within the family?	How did you feel when you heard your parent was ill?	What does your family look like?
Autonomy	Are your children able to live their own life? What helps this? What limits this? Has this changed? What would help for more balance?	Do you find it difficult to do your own things when your parent is ill? Do you feel you have the freedom to do so? What limits you? Has this changed? What would be a good balance? What would help?	Do you find it difficult to live your own life when your parent is ill? What does it mean to you to feel free? How do you ensure this? What limits you? Has this changed since the illness? What would be a good balance?
Connectedness	What is the relationship like with your child(ren)? When do you feel connected? Has this changed since the illness?	What is the relationship like with your parents and siblings? When do you feel connected? What helps you feel connected? Has this changed since the illness?	What helps you feel “together” as a family? What could help even more?
Communication	What is communication like within your family? Has this changed since the illness? Are there things you do not discuss? What helps to be open? What could help?	Same as parent interview	Is your family one that easily or hardly discusses things? Has this changed since the illness? Are there things you do not discuss? What helps to be open? What could help?
Closing	Is there anything else you’d like to discuss that we haven’t already discussed? Do you have any advice for other families?	Same as parent interview	Same as parent interview

### Data analysis

Interviews were recorded and transcribed verbatim for analysis by author GZ and a research assistant. The interviews were subsequently analyzed in ATLAS.ti (Version 24) using inductive thematic analysis following the phases outlined by Braun and Clarke [13]. Within the topics of autonomy, communication, and connectedness, coding was inductive. The first six interviews were independently coded by GZ and HB, with discrepancies discussed until consensus was reached. Codes were then reviewed with ML and MS and adjusted accordingly. This led to a coding framework where codes consisted of (1) the main topic, (2) family member, and (3) an inductive summary. Additional topics (coping, emotions, and tips) emerged during coding. The remaining interviews were coded by GZ. Themes were generated stepwise with all authors, discussing the codes and their underlying quotes, merging similar codes, and refining and defining the finalized themes and subthemes. Finally, the manuscript was written according to COREQ reporting guidelines [14].

## Results

### Sample characteristics

A total of 21 interviews were conducted with 8 families: 7 with parent(s), 7 with child(ren), and 7 with the whole family (see Table 3 for the family-level clinical characteristics). Notably, in family G, only a joint interview was held, whereas in family B, only separate interviews were conducted. Data collection ended upon thematic saturation. The sample consisted of families where at least one parent had been diagnosed with cancer, most commonly breast cancer (55.6%). The time since diagnosis ranged from 27 months to 11 years. The majority of ill parents were women (75%). For full sociodemographic and clinical characteristics of the 27 participants, see Table 2.

**Table 2:** Sociodemographic and clinical characteristics of the 27 participants

	**Ill parent (*n* = 9)**^**a**^	**Non-ill parent (*n* = 4)**	**Children (*n* = 14)**
Age, *M* (SD)	54.3 (5.8)	54 (8.2)	21.7 (4)
Gender, *n* (%) Man Woman	2 (22.2) 7 (77.8)	3 (75) 1 (25)	8 (57.1) 6 (42.9)
Educational level, *n* (%) Low Intermediate High In school	- 2 (22.2) 7 (77.8) -	- 2 (50) 2 (50) -	- 2 (14.3) 2 (14.3) 10 (71.4)
Working status, *n* (%) Employed Disabled	7 (77.8) 2 (22.2)	4 (100) -	-
Diagnosis Breast cancer Multiple myeloma Leukemia Sarcoma Brain tumor	5 (55.6) 1 (11.1) 1 (11.1) 1 (11.1) 1 (11.1)		
Treatment received Surgery Chemotherapy Radiotherapy Hormone therapy Immunotherapy Targeted therapy Stem cell transplant	7 (77.8) 7 (77.8) 6 (66.7) 4 (44.4) 3 (33.3) 1 (11.1) 1 (11.1)		
Time since last treatment In treatment 6–12 months 1–2 years > 2 years	1 (11.1) 1 (11.1) 2 (22.2) 5 (55.6)		

Note. ^a^There were 9 ill parents across 8 families, as one family included 2 ill parents

### Thematic analysis

The thematic analysis yielded three main themes: (1) children’s struggle between personal space and family life, (2) barriers and opportunities in family communication, and (3) redefining connectedness. Several subthemes emerged from the data. These (sub-)themes describe the challenges of families with parental cancer, who deal with balancing facing the disease and maintaining a sense of normality as a family (Table 3). The themes and their subthemes are outlined in the sections below. For additional quotes supporting the themes, see the supplementary material.

**Table 3:** Clinical characteristics of the 8 families

**Family**	**Patient**	**Parents interviewed**	**Parental living situation**	**Children interviewed**	**Child age(s)**^**a**^	**Time since diagnosis in months**	**Treatment intent**
A	Father	2	Married/living together	2/2	21,18	29	Non curative
B	Mother	2	Married/living together	2/2	23,20	130	Non curative
C	Both (father died)	1	Widowed	1/2	26 (29)	53	Curative
D	Mother	1	Divorced	1/3	19 (21 + 17)	29	Curative
E	Both	2	Married/living together	2/3	29, 26 (31)	168 (mother), 151(father)	Curative (both)
F	Mother	1	Single	1/3	21 (18 + 11)	44	Curative
G	Mother	2	Married/living together	2/4	20, 17 (12 + 7)	84	Non curative
H	Mother	2	Married/living together	1/2	17 (14)	27	Curative

Note. ^a^Parentheses indicate children who did not participate in the interviews

### Theme 1: children’s struggle between personal space and family life

Children described a continuous struggle between their need for personal space and their sense of responsibility towards their family.

#### Looking for normalcy outside the home

Some children consciously seek an environment outside the family, such as engaging in sports activities, to momentarily step away from the illness at home. Stepping away from the role of ‘the child of a parent with cancer’ can bring a sense of relief, allowing them to participate in activities that restore a feeling of normalcy.

*I had a gap year after high school and I liked being completely out there for a while and I also liked getting to know a whole new group of people there with whom you are not always that kid with the two parents with cancer.* – Family E, child, 26

#### Feeling guilty about living their own life

For children, taking their own space often comes with feelings of guilt, especially when they spend time away from home. For example, they feel the need to check in daily with their ill parent. Parents’ reaction could further increase these feelings of guilt and pressure them to spend more time at home:

*Sometimes I find it difficult, like when I go to have dinner at my best friend’s house in the evening. For example, they’ll say something like, ‘Oh, off to [friend] again? Must be nice.’ Comments like that get to me because they amplify the guilt I already feel. It makes me feel ten times worse. I still go, but I leave with a heavy heart. So, I’m still trying to find the right balance in that. –* Family B, child, 20

#### Feeling responsible for family duties

Many children feel an increased sense of responsibility within the household, especially in single-parent families, where the absence of another adult increases the pressure to help. This can mean taking over household chores, caring for siblings, or supporting the ill parent emotionally.

*I don’t have many memories from the period after my mom’s diagnosis, because I shifted into a role of providing as much support as possible. I took on a lot of household responsibilities so that my father wouldn’t have to do too much, and I also tried to shield my younger sister from it, so she could have a relatively normal childhood. I didn’t want to take the joy away from her. – *Family B, child, 23

#### Consciously choosing family time

While many children struggle between being present and seeking space, some choose to spend more time with their ill parent. A poor prognosis increases their awareness of limited time, leading them to stay closer to home. One child reflects on this dilemma:

*Sometimes we all have dinner, talk and laugh together. And then I think: I won’t have that once I move out. And what if I move out, and six months later she passes away? Won’t I regret missing out on those moments? –* Family B, child, 23

### Theme 2: barriers and opportunities in family communication

Communication within families was often limited, especially when it came to expressing emotions. This theme explores both the barriers that hinder open dialogue as well as moments when emotional connection was possible.

#### Non-verbal communication

Families do not always need words to understand each other. Participants described how, upon hearing the diagnosis, they cried together and embraced one another. Family members are also often able to sense each other’s emotions without speaking. They pick up on non-verbal cues such as facial expressions, which can signal that something is wrong and trigger a sense of concern or alertness.

*I clearly remember my mom picking me up from school, and even just seeing her face from the bus, I already knew something was wrong. I had no idea what, but I could tell. –* Family E, child, 29

#### Limited communication in survival mode

During diagnosis, treatment, or disease progression, families entered a 'survival mode':

*I think we were all in a kind of survival mode, just trying to get through this period. You don’t reflect, you just keep going: surgeries, appointments, treatments. You push forward.* – Family D, ill mother, divorced, 3 children

In this mode, there was little space for reflection or communication. Parents focused on the bare minimum of keeping things going, while children faced their usual challenges at school and in social life, now compounded by the strain of changing family dynamics.

#### Not wanting to burden others with feelings

Participants shared little of their own worries and emotions related to the cancer with family members. Children tended to keep their feelings to themselves, believing their parents’ suffering was more severe and their own struggles less important.

*I can’t remember a time when, while she was ill, you would say something like, 'I feel terrible too' or anything like that. Not that it wasn’t possible, but you just don’t do that easily. After all, she’s the one who’s ill, and that’s obviously much worse.* – Family F, child, 21

This norm made it harder for parents to understand how their children were truly feeling. At the same time, parents did not set an example of openness either, as they avoided sharing their own concerns in an attempt to shield their children and preserve a sense of normalcy.

#### Being active together supports communication

Spontaneous moments, like driving, walking, or biking together, make it easier for family members to talk, as conversations arise naturally and feel less heavy.

*I remember that I usually brought things up while cycling. I just found it easier. On the bike, you must look ahead, so you don’t have to make eye contact. If we had been sitting face-to-face, it might have felt a bit awkward. –* Family A, child, 18

Parents described how they actively create moments of connectedness, for example, by going on holiday with a child or having lunch or sports together, making an effort to spend one-on-one time with their child to keep it easy for conversation to occur.

#### Focus on practicalities of emotionally charged issues

Emotional topics were often difficult to discuss, while practical matters were addressed more easily. Families coordinated hospital visits, shared updates, or arranged household responsibilities together.

*As long as we communicated clearly, everything was fine. My sister and I used a group chat to set a schedule for household tasks. It was all very practical, not really about emotions. But it brought calm and let everyone focus on their own activities. –* Family D, child, 19

Some families also talked about funeral wishes such as burial or cremation, music choices, or the handling of ashes, with conversations remaining practical, rather than discussing the emotions involved.

### Theme 3: redefining connectedness

Since the illness, families noted an increased sense of connectedness. The presence of cancer required new strategies for maintaining this bond. Families developed various ways to foster connectedness.

#### The medical process as a shared family experience

Children differed in the extent to which they were involved in the parent’s illness. Some parents actively included their children in the medical process, for example, by taking them to medical appointments or involving them in choosing a grave.

*My daughter came with me when I went to pick out a wig. Those were intense moments, but sharing them together helped us stay connected. –* Family F, ill mother, single, 3 children

On the other hand, some parents chose not to involve their children in the medical process to avoid burdening them with the weight of caregiving. In addition, some children preferred to distance themselves from their parents’ medical process.

#### Recognizing and responding to each other’s needs

When family members are able to sense and respond to each other’s needs, it can strengthen their sense of connectedness. This alignment does not always require words. Being emotionally aligned can offer comfort and support during difficult times, and can strengthen the connection between parent and child.

*I think that sometimes the feeling of connection came more from experiencing intense moments together. For example, there was a day when my mother wasn’t feeling well because of the chemo, and we spent the whole day lying down and listening to music together. Those are memories that have really stayed with me and created a sense of connection, but in a completely different way than, for example, doing sports together for fun.* – Family E, child, 26

Family members were sometimes unaware of each other’s needs. They may miss signals or hesitate to act, unsure of how to be there for one another, which can lead to moments of distance or silence.

*At one point, I was lying upstairs while the three of them were here (downstairs) together. I think it was the first or second day (since chemotherapy had started - GZ). They were sitting here together, enjoying each other’s company, but at the same time, they didn’t dare to leave. But they also didn’t dare to come upstairs to ask, ‘Mom, how are you doing?’ because they assumed I was asleep. –* Family D, ill mother, divorced, 3 children

#### Continuing to enjoy quality time together despite limitations

Engaging in enjoyable activities helped families maintain cohesion. Despite cancer-related limitations, parents found new ways to spend quality time, preserving a sense of connection.

*I regularly go to Ajax* *(Dutch football club - GZ)* *with the boys, so we were watching football instead of playing football like we did before. Well, it’s not exactly a big adventure. But you do have a moment together as a family. –* Family E, ill father, 3 children

#### Humor as a family coping

For family members, dealing with cancer and stressful situations is challenging. Humor can be a way to make situations less stressful and serious, while still acknowledging the presence of cancer.

*I’m very happy that we have that humor with each other, that we can really laugh at things, even though those things were sometimes very miserable. That takes the sting out of it for a while. –* Family F, ill mother, single, 3 children

## Discussion

The present study explored how families with AYAs balanced autonomy and connectedness during parental cancer and the role of communication in this. Some children consciously choose to spend time with their family, seeing it as an autonomous choice. They could search for normalcy outside the house, while others struggled with the balance of feeling responsible for the family and feeling guilty for living their own life. Children’s struggles with autonomy were less present in families with strong connectedness. Sharing the medical process as a family contributed to this sense of connectedness. One important factor in fostering connectedness was emotional attunement. When family members sensed each other well, the lack of open communication about illness-related difficulties was not problematic. In families lacking this emotional attunement, the lack of open communication resulted in a greater distance and more misunderstandings between the child and the parent. Being active together (e.g., cycling together) could then facilitate emotional conversations.

In Walsh’s framework, family processes are understood as dynamic and synergistic, amplifying each other [11]. In the present study, we observe a similar process between autonomy, connectedness, and communication. Some families reported a strong balance between autonomy and connectedness. Children felt closely connected to their family while simultaneously experiencing autonomy in making their own choices. Despite limited explicit communication, they were attuned to one another and experienced connectedness through implicit understanding. In well-functioning families, we see that autonomy, connectedness, and communication all reinforce each other positively.

In contrast, there were families in which limited communication coincided with uncertainty about each other’s needs and avoidance of emotionally charged conversations. This resulted in a reduced sense of connectedness. Because children were not open about their own needs, they could experience feelings of guilt when they needed space for themselves. This complicated the balance between autonomy and connectedness. Previous research also found that children struggle with this balance, describing that young carers alternate between wanting to care and wanting to escape [15]. Looking for normalcy outside their own home is essential for the wellbeing of adolescents and young adults, though some struggle with feelings of guilt when choosing their own life over that of the family. When this balance between connectedness and autonomy is disrupted, the lack of connectedness, communication, and autonomy also amplifies, creating a negative vicious circle.

The amplifying patterns described above are also seen in couples coping with cancer, where protective buffering (withholding personal concerns to avoid burdening the other) is associated with decreased relationship functioning [16], while open communication supports stronger relationships [17].

### Limitations and strengths

Nearly 70% of participants were highly educated, limiting generalizability. In addition, most ill parents were female, which limits our understanding of families with fathers who have cancer. For future research, more diverse samples should be included. Moreover, children’s ages were recorded at the time of interview, not during illness, meaning the cancer trajectory could have occurred years earlier, potentially affecting recall and reflecting a different developmental stage. Nevertheless, the joint family interview captured shared reflections and real-time dynamics, while the individual interviews allowed parents and children to share their perspectives without influence from other(s). Together, these provide a valuable source of information. Future research could build upon this study by using in-the-moment approaches such as experience sampling to capture interaction patterns within families.

### Practical implications

Our findings suggest several ways professionals can support families living with parental cancer. In hospital settings, nurses can play a preventive role by considering the needs of the entire family, not only the patient, and by actively involving children when they wish to participate, for example, by providing medical information in age-appropriate ways. Professionals should also help families in strengthening existing emotional bonds and help foster mutual understanding and emotional attunement. When emotional attunement is lacking and family members appear unable to understand each other, referral to a systemic therapist may be required. In addition, these insights offer practical guidance for families themselves, such as maintaining meaningful activities, doing activities that foster open communication, using humor, and involving children in the medical process to support family resilience.

## Conclusion

This study investigated how families need to find new ways to navigate the balance between autonomy and connectedness, and communication when confronted with parental cancer.

In some families, strong emotional attunement appeared to make navigating autonomy and connectedness natural. Even though open communication was limited, this did not appear problematic. Other families struggled more with these dynamics. Practical implications include continuing meaningful family activities - adapted to the limitations of illness, doing activities that foster open communication, using humor, and involving children in the medical process. When emotional attunement does not arise naturally, supportive interventions such as family therapy may help strengthen this.

## Supplementary Information

Below is the link to the electronic supplementary material.

ESM 1 (DOCX 29.7 KB)

## Data Availability

The datasets generated and/or analyzed in the current study are not available publicly as eligible patients were informed before start of the interviews that their data would be stored securely and confidentially.
