# Supplementary material for: Navigating family life in the face of parental cancer: a qualitative study
Source: Support Care Cancer. 2026 Apr 22;34(5):454. doi: 10.1007/s00520-026-10670-6 (PMC13102880; doi:10.1007/s00520-026-10670-6)
Supplement: Supplementary file 1 — (DOCX 29.7 KB) [file 520_2026_10670_MOESM1_ESM.docx]

**Table 1**

*Additional Quotes Supporting the Themes*

| **Theme 1: Children’s struggle between personal space and family life** | |
| --- | --- |
| Looking for normalcy outside the home | *‘I play [sport] at a high level in my free time, so skipping training wasn’t really an option. I could have if my mom wasn’t doing well, but I just kept going. It was a way for me to have my own space for a while.’*   - Family F, child, 21   ‘*I had a gap year after high school and I liked being completely out there for a while and I also liked getting to know a whole new group of people there with whom you are not always that kid with the two parents with cancer.*’   - Family E, child, 26 |
| Feeling guilty about living their own life | *‘I remember one of the first days I went back to school after dad’s diagnosis, I said: ‘I find it hard to tell now, but I actually had quite a nice day’. Because you kind of feel guilty that you did have a nice day while they only had shitty days then. And that I could then go away and have a nice day there for a while.’*   - Family A, child, 21   *‘I had a sense of guilt because I was spending time in my student house. In the beginning, I stayed at home a lot, even though I already had this room. Still, I felt the need to check in every day, asking, ‘How are you feeling today? Is there anything I can do? Should I come by?’ And my mom would always say: ‘No, don’t worry, go do your own thing.’ But that still felt a bit strange.’*   - Family F, child, 21   *‘Sometimes I find it difficult, like when I go to have dinner at my best friend’s house in the evening. For example, they’ll say something like, ‘Oh, off to [friend] again? Must be nice.* (sarcasm, GZ)*’ Comments like that get to me because they amplify the guilt I already feel. It makes me feel ten times worse. I still go, but I leave with a heavy heart. So, I’m still trying to find the right balance in that.’*   - Family B, child, 20 |
| Feeling responsible for family duties | *‘I don't have many memories from the period after my mom’s diagnosis, because I shifted into a role of providing as much support as possible. I took on a lot of household responsibilities so that my father wouldn't have to do too much, and I also tried to shield my younger sister from it, so she could have a relatively normal childhood. I didn’t want to take the joy away from her.’*   - Family B, child, 23   *‘Of course, there’s also household responsibilities, like who’s going to cook and who’s going to take care of things. Because of that, you end up spending more time at home. You eat together more often instead of deciding to have dinner with friends, because if mom is home, you feel inclined to stay and eat at home instead.’*   - Family D, child, 19   *‘For the children, it was difficult to lead their own lives because they also took on the responsibility of caring for me. I think they regained their sense of freedom about three months after my last surgery, once I was able to do everything on my own again.’*   - Family D, mother |
| Consciously choosing family time | *‘I think that, especially in the beginning, I really wanted to support my parents more, so I stayed home more often. And after that, I just continued to spend a lot of time at home, simply because I enjoy being there and spending time with them.’*   - Family A, child, 21   *‘Sometimes we* (refers to her family, GZ) *have an evening where the four of us, or just three of us, are at home, and we watch a movie together or play a game. We always have dinner together at the table in the evening, just having small conversations and laughing. And then I think, yeah, I won’t have that once I move out. And what if I move out, and six months later she passes away? Won’t I regret missing out on those moments for those six months?’*   - Family B, child, 23 |
| **Theme 2: barriers and opportunities in family communication** | |
| Non-verbal communication | *‘I still remember, that was the first time I saw my father cry, and it was really intense for me. But I also remember that afterwards we had a really comforting moment, hugging each other.’*   - Family B, child, 20   *‘I clearly remember my mom picking me up from school, and even just seeing her face from the bus, I already knew something was wrong. I had no idea what, but I could tell.’*   - Family E, child, 29   *‘In the beginning, we cried about it together a lot. It was very confronting to realise that I had cancer. (…) And in a way, we were forced to grow emotionally closer, and that actually turned out to be something beautiful.’*   - Family B, ill father |
| Limited communication in survival mode | *‘I think we were all in a kind of survival mode, just trying to get through this period. That’s why, in hindsight, you really need to take a moment to reflect, because during that survival mode, you don’t. You just keep going because you have to - getting through the surgeries, the appointments, the treatments - you just push forward.’*   - Family D, ill mother   *‘When she* (refers to mother, GZ) *switched to the pills and was no longer in the hospital, the hospital bed downstairs was gone, and life started to feel a bit more normal again. And I think that’s when it really hit me. Like I finally had some mental space to actually think about everything and to reflect on what it had done to me,’*   - Family B, child, 23   *‘Talking... how much did I really talk? On one hand, we* (as a family, GZ) *did talk about it, but it was often very general. At some point, you just don’t feel like talking anymore. You start thinking, ‘Please, everyone, just stop asking. I don’t want to talk about it. And honestly, I don’t even know how I feel myself.’ I had one close friend I shared a lot with, but beyond that, I just didn’t want to. The idea of updating 40 people about everything was too much. I didn’t have the space for that.’*   - Family C, child, 26 |
| Not wanting to burden others with feelings | *‘I can’t remember a time when, while she was ill, you would say something like, ‘I feel terrible too’ or anything like that. Not that it wasn’t possible, but you just don’t do that easily. After all, she’s the one who’s ill, and that’s obviously much worse.’*   - Family F, child, 21   *‘My son is also quite reserved. You really have to look for signs to understand what he’s feeling. I try to observe how he’s doing, whether he’s struggling with it, and things like that. But it’s difficult… I just can’t seem to get it out in the open. He shares very little, and I’m not sure if he doesn’t tell me because he doesn’t want to hurt me or if he simply doesn’t want to talk about it. Maybe because he doesn’t want to confront his own feelings. I just don’t know. It’s a bit difficult.’*   - Family H, ill mother   *‘But as a parent, you don’t want to share your concerns and feelings with your child, because, well, I didn’t want to burden them either.’*   - Family H, ill mother   *‘I feel like it’s just repeating the same struggles over and over. I’ve dealt with so much, like endless hormone treatments after chemo, which affected my mood and everything else. But I’m also just getting older, and I think, ‘Does it even matter to them?’ That’s why I tend to share these things with my friends rather than my children. I don’t want to burden them with all that.’*   - Family E, ill mother   *‘I noticed in myself that sometimes I just really wanted to see my parents acknowledge that this situation just sucks. I think they tried to protect us from those really tough moments. Of course, you notice when things aren’t going well, but looking back, I do feel like it was rarely actually discussed. In hindsight, it would’ve been nice to sometimes just talk about how awful everything really was and how we were all experiencing it, instead of ‘no worries, everything’s fine, go enjoy your sports and whatever.’*   - Family E, child, 29 |
| Being active together supports communication | *“I remember that I usually brought things up while cycling. I don’t know why, but I just found it easier. On the bike, you have to look ahead, so you don’t have to make eye contact. If we had been sitting face-to-face, it might have felt a bit awkward.”*   - Family A, child, 18   *"You have to see each other regularly to even create those moments. We also try to make space for one-on-one time, like how you* (her husband, GZ) *always take our son to football. I often remind you to ask him about certain things during those moments. We really try to create those opportunities because, of course, you can’t just dive into deep conversations every time you see each other. You need enough everyday contact first."*   - Family E, ill mother |
| Focus on practicalities of emotionally charged issues | *‘I was fine with being cremated. But I thought: let’s talk about it as a family too, because I won’t be here anymore, and I believe you all should have a say in it. And we didn’t agree as a family. One of them wanted a burial, to have a place to return to, while another wanted to do something with the ashes, like having them made into a ring or something.’*   - Family A, ill father     *‘You really have to make sure to tell them when check-ups are coming. We’ve tried not mentioning it before and then just saying afterwards, ‘It was all fine,’ but the kids don’t really like that. So now we do tell them, but by now it’s been so long that it’s more of an announcement. I don’t think they really lose sleep over it anymore.’*   - Family E, ill mother   Emotional topics were often more difficult to discuss:  *‘Speaking for myself, I do feel that, both personally and as a family, it was quite difficult at times to really get to the core of things. Like, how are we feeling? What’s really going on with you? Or how do you really feel about all of this?’*   - Family E, child, 29 |
| **Theme 3: Redefining connectedness** | |
| The medical process as a shared family experience | *‘My daughter came with me when I went to pick out a wig. Those were intense moments, of course, but sharing them together helped us stay connected. At the same time, it was also important to give each other space when one of us didn’t feel like talking about it.’*   - Family F, ill mother   *‘Before I started chemotherapy, the children were allowed to come and see where it all would take place, so we visited [the hospital]. We showed them: this is the room where I’ll receive the chemo, and this is the ward where I’ll be admitted. We were allowed to look everywhere, and the children also wrote a letter to the doctor. That was a really good way for them to feel involved.’*   - Family G, ill mother   *‘I also went with her to the hospital. I attended many appointments, and then of course you immediately talk about these things. But I think that only strengthened our bond.’*   - Family F, child, 21   On the other hand, some parents chose not to involve their children in the medical process to avoid burdening them with the weight of caregiving. In addition, some children preferred to distance themselves from their parents’ medical process.  *‘I immediately felt that it was already difficult enough. The children shouldn’t have to become caregivers, taking on the role of escorting me to the hospital.’*   - Family C, ill mother   *‘I didn’t really go with her to the hospital, because I didn’t feel comfortable there. You see other people who are very ill, or sicker than my mother is, and then you start thinking: maybe my mother will end up looking that sick too, and that’s not a comforting image.’*   - Family H, child, 17 |
| Recognizing and responding to each other’s needs | *‘At one point, I was lying upstairs while the three of them were here (downstairs) together. I think it was the first or second day* (since chemotherapy had started, GZ). *They were sitting here together, enjoying each other’s company, but at the same time, they didn’t dare to leave. But they also didn’t dare to come upstairs to ask, “Mom, how are you doing?” because they assumed I was asleep.’*   - Family D, ill mother, divorced, 3 children   *‘I think that sometimes the feeling of connection came more from experiencing intense moments together. For example, there was a day when my mother wasn’t feeling well because of the chemo, and we spent the whole day lying down and listening to music together. Those are memories that have really stayed with me and created a sense of connection, but in a completely different way than, for example, doing sports together for fun.’*   - Family E, child, 26 |
| Continuing to enjoy quality time together despite limitations | *‘I regularly go to* (Dutch football club, GZ) *with the boys, so we were watching football instead of playing football like we did before. Well, it’s not exactly a big adventure. But you do have a moment together as a family.’*   - Family E, ill father, 3 children   *‘We heard the diagnosis while we were in the car on our way to our vacation. The vacation was a little less enjoyable because of that, but we still managed to make it a good one. We just wanted to spend some nice time together.’*   - Family H, child, 17   *‘I was moving to my student room right when my mother had just become ill, which was really bad timing. She wasn’t allowed to drive at that point, but she would still look online for furniture and things like that. Then we’d spend a day setting everything up. We would assemble and arrange everything, while she did something at the table.’*   - Family F, child, 21   Father: *‘During the first year after my surgery, we went on vacation and ended up climbing some kind of volcano. I had my crutches with me, but the kids all helped me get up and down that thing.’* Mother*: ‘It was really nice, it really felt like a family moment.’*   - Family E, ill father and ill mother, 3 children |
| Humor as a family coping | *‘I'm very happy that we have that humor with each other, that we can really laugh at things, even though those things were sometimes very miserable. That takes the sting out of it for a while.’*   - Family F, ill mother, single, 3 children   *‘We do joke around a lot together, and that really connects us. Sometimes the humor can get a bit rough, and then my son says something a little on the crude side. And then we still end up laughing about it together.’*   - Family H, ill mother, 2 children   *‘We tried to keep things light during those years when we were ill. We were also quite often joking around. I know that with my dad, for example, we were joking about things like: if you lose half your leg anyway, can’t they attach something underneath so you can still go skiing?’* (family laughs together, GZ)   - Family E, child, 29 |
